# The Efficacy of Montelukast in Reducing the Risk of Warning Signs and Subsequent Dengue Shock Syndrome in Adult Dengue Fever Patients

**DOI:** 10.7759/cureus.61603

**Published:** 2024-06-03

**Authors:** Anand Dev, Raj B Singh, Nitali Arun, Shivani Sinha

**Affiliations:** 1 General Medicine, Indira Gandhi Institute of Medical Sciences, Patna, IND; 2 Anesthesiology, Indira Gandhi Institute of Medical Sciences, Patna, IND; 3 Microbiology, Radha Devi Jageshwari Memorial Medical College and Hospital, Muzaffarpur, IND; 4 Community Medicine, Indira Gandhi Institute of Medical Sciences, Patna, IND

**Keywords:** therapeutic intervention, randomized controlled trials, dengue shock syndrome, montelukast, dengue fever

## Abstract

Background: Dengue fever continues to pose significant health challenges globally, with recent outbreaks in Bihar, India, prompting a search for effective therapeutic interventions. This study assesses the effectiveness of Montelukast, traditionally used for asthma, in mitigating the severity of dengue fever symptoms and its progression to dengue shock syndrome (DSS).

Objective: To evaluate the impact of Montelukast on the prevalence of dengue warning signs and the incidence of DSS in adult patients.

Methods: A prospective observational study was conducted at the Indira Gandhi Institute of Medical Sciences (IGIMS), Patna, India, from August 2022 to October 2023, enrolling 500 patients diagnosed with dengue fever. Participants were divided into two groups. About 250 were treated with Montelukast and 250 received standard care. Outcomes measured included the incidence of warning signs, DSS, length of hospital stay, and 30-day mortality.

Results: The Montelukast group exhibited a 24% lower prevalence of dengue warning signs compared to the control group, with 90 out of 250 patients (36%) in the Montelukast group versus 150 out of 250 patients (60%) in the control group (p < 0.001). The incidence of DSS was significantly reduced in the Montelukast group, with 4 out of 250 patients (1.6%) compared to 21 out of 250 patients (8.4%) in the control group (odds ratio: 0.178, p < 0.001). Furthermore, Montelukast users experienced shorter hospital stays (average 4.52 days vs. 6.54 days, T-statistic: -7.59, p = 1.58×10^-13^) and a reduced 30-day mortality rate, with 5 out of 250 patients (2%) in the Montelukast group versus 12 out of 250 patients (5%) in the control group (p < 0.03).

Conclusion: Montelukast significantly lowers the incidence of dengue warning signs and DSS, shortens hospital stays, and decreases mortality rates among dengue patients, supporting its potential integration into existing dengue treatment protocols. This study highlights the need for further clinical trials to confirm these findings and fully understand the therapeutic mechanisms of Montelukast in dengue management.

## Introduction

Dengue fever is a mosquito-borne viral infection that continues to pose a considerable threat to global health [1]. Transmitted by the Aedes mosquito species, the dengue virus (DENV) is responsible for infecting millions worldwide, with India being one of the most severely affected countries. Unexpected outbreaks in the year 2023 in previously unaffected regions, such as Bihar, have led to a surge in hospitalizations and mortality rates. As per the latest reports, Bihar was left grappling with a severe dengue outbreak in 2023, with more than 15,000 reported cases and 59 deaths [2]. This marks the highest number of dengue cases and fatalities in the state in the last five years, signifying a remarkable 608% increase in cases compared to 2018. Despite the grave implications, the medical community still grapples with a lack of specific antiviral treatments for dengue, relying heavily on symptomatic and supportive care. This deficiency underscores the urgency to discover effective therapeutic interventions to curtail the severity and subsequent complications tied to the disease.

Role of mast cells in dengue pathogenesis

Central to the discussion on dengue's severity is the role of mast cells. These cells are crucial in steering innate immune responses. Their activation culminates in the release of several agents like cytokines, histamine, heparin, and notably, leukotrienes. The latter has gained attention for its significant role in promoting plasma leakage and leukocyte adhesion, both of which are critical in instigating severe vascular complications in dengue patients [3,4].

Montelukast: a potential therapeutic agent

Montelukast, a recognized leukotriene inhibitor, has emerged as a beacon of hope in this context. Primarily utilized for managing respiratory ailments such as asthma, Montelukast has exhibited potential in animal studies by curbing plasma leakage and minimizing dengue-associated vascular complications [5-7]. Preliminary clinical evaluations have further supported this, showcasing a notable decline in dengue shock syndrome (DSS) incidences upon Montelukast treatment. Yet, the absence of a comprehensive study evaluating Montelukast's efficacy in dengue patients remains a significant gap in the existing research.

Implications of dengue outbreaks

Beyond the immediate health implications, dengue outbreaks exert immense pressure on healthcare infrastructures, amplifying hospitalizations, straining resources, and leading to economic repercussions. Thus, uncovering an effective intervention, like Montelukast, that can diminish the risk of severe dengue manifestations can radically transform public health paradigms, enhance patient outcomes, and optimize healthcare resource management.

Against this backdrop, our study aims to conduct a rigorous observational study to investigate the associations between Montelukast use, the prevalence of warning signs, and DSS in adult patients diagnosed with dengue fever.

Aims and objectives

The primary goal of this study is to examine the associations between Montelukast use and the prevalence of dengue warning signs and DSS among adult patients diagnosed with dengue fever. To ensure a comprehensive assessment of Montelukast's therapeutic potential, the study focused on key outcomes including the rate of dengue with warning signs, the rate of DSS, the length of hospital stay, and the 30-day mortality rate.

## Materials and methods

Study design

This study was designed as a prospective observational study, aiming to provide a rigorous evaluation of the associations between Montelukast use and key clinical outcomes in dengue.

Setting

The study was meticulously conducted within the premises of the Emergency and General Medicine Departments of Indira Gandhi Institute of Medical Sciences (IGIMS), Patna, India, from August 1, 2022, to October 15, 2023.

Study arms

Participants in the study were categorized into two distinct groups based on their treatment history. The Montelukast users group included patients who were prescribed a 10 mg Montelukast tablet orally daily as part of their routine clinical care. The non-Montelukast users' group comprised patients who were not administered Montelukast and received standard care for dengue.

Participant criteria

The study enrolled a total of 500 adult participants, each being at least 18 years of age. A confirmed diagnosis of dengue, indicated by a positive NS1 antigen or enzyme-linked immunosorbent assay (ELISA) for dengue, was a prerequisite for inclusion in the study.

Inclusion criteria

To be eligible for the study, participants had to be adults aged 18 or older with a confirmed diagnosis of dengue, as indicated by a positive NS1 antigen or ELISA for dengue.

Exclusion criteria

Participants were excluded from the study if they had any contraindications for Montelukast, a concurrent diagnosis of other fever-related conditions like malaria or heat stroke, were pregnant, had critical illnesses that required intensive care, were unable to intake oral medication, or faced communication barriers or challenges.

## Results

Montelukast users showed a 24% lower prevalence of warning signs, with 90 out of 250 patients (36%) in the Montelukast group versus 150 out of 250 patients (60%) in the non-Montelukast group, with significant reductions in symptoms like persistent vomiting, 15 out of 250 patients (6%) in the Montelukast group versus 35 out of 250 patients (14%) in the non-Montelukast group; abdominal pain, 20 out of 250 patients (8%) in the Montelukast group versus 45 out of 250 patients (18%) in the non-Montelukast group; and elevated hematocrit levels, 15 out of 250 patients (6%) in the Montelukast group versus 40 out of 250 patients (16%) in the non-Montelukast group. The incidence of DSS was notably lower among Montelukast users, with 4 out of 250 patients (1.6%) compared to 21 out of 250 patients (8.4%) in the non-Montelukast group, as evidenced by an odds ratio of 0.178. Montelukast users experienced shorter hospital stays, with an average of 4.52 days compared to 6.54 days for non-Montelukast users, a finding supported by a highly significant t-statistic of -7.59. The mortality rate within 30 days was halved in the Montelukast group, with 5 out of 250 patients (2%) compared to 12 out of 250 patients (5%) in the non-Montelukast group. Kaplan-Meier Survival Curve analysis further supported this finding.

Table 1 provides a comprehensive demographic and clinical profile of the study population, consisting of 500 participants equally divided between the Montelukast group and the non-Montelukast group. It details key characteristics such as age, gender distribution, location, socioeconomic status, education level, household size, travel history, access to healthcare facilities, local climate conditions, and diagnostic methods. The data indicate comparable demographics and baseline health statuses across both groups, ensuring a balanced comparison in subsequent analyses of Montelukast's effects on dengue outcomes.

**Table 1:** Demographic and clinical characteristics of the study population ^*^Serum NS1 antigen positive: non-structural protein 1 antigen positive ^#^ELISA for dengue positive: enzyme-linked immunosorbent assay for dengue positive The age and household size values are represented as mean ± standard deviation (SD). Other values are represented as the number of patients (percentage of the group).

Characteristic	Montelukast group (n = 250)	Non-Montelukast group (n = 250)
Age (years)	35 ± 10	36 ± 9
Gender		
- Male	150 (60%)	145 (58%)
- Female	100 (40%)	105 (42%)
Location		
- Urban	150 (60%)	155 (62%)
- Rural	100 (40%)	95 (38%)
Occupation		
- Low socioeconomic status	85 (34%)	90 (36%)
- Moderate socioeconomic status	100 (40%)	95 (38%)
- High socioeconomic status	65 (26%)	65 (26%)
Education level		
- Primary	70 (28%)	75 (30%)
- Secondary	110 (44%)	100 (40%)
- Higher	70 (28%)	75 (30%)
Household size (mean ± SD)	4.5 ± 1.8	4.6 ± 1.7
Recent travel history		
- Yes	50 (20%)	55 (22%)
- No	200 (80%)	195 (78%)
Access to healthcare facilities		
- Proximity	180 (72%)	170 (68%)
- Distance	70 (28%)	80 (32%)
Local climate and seasonality		
- Monsoon	130 (52%)	135 (54%)
- Non-monsoon	120 (48%)	115 (46%)
Diagnosis method		
- Serum NS1 antigen positive^*^	150 (60%)	145 (58%)
- ELISA for dengue positive^#^	100 (40%)	105 (42%)

Table 2 presents a comparative analysis of the incidence of various dengue warning signs between the Montelukast and non-Montelukast treatment groups. This table details the frequency and percentage of patients who experienced each warning sign within the respective groups, along with the statistical significance of these occurrences.

**Table 2:** Rate of dengue with warning signs *indicates statistical significance

Warning signs	Montelukast group (n = 250)	Non-Montelukast group (n = 250)	95% confidence Interval (CI)	p-value
Total with warning signs	90 (36%)	150 (60%)	0.18 to 0.28	<0.001*
Persistent vomiting	15 (6%)	35 (14%)	0.04 to 0.12	0.0046*
Pain abdomen	20 (8%)	45 (18%)	0.06 to 0.14	0.0014*
Serositis	15 (6%)	35 (14%)	0.04 to 0.12	0.0046*
Bleeding from mucosal sites	10 (4%)	25 (10%)	0.02 to 0.10	0.0141*
Increase in hematocrit	15 (6%)	40 (16%)	0.04 to 0.14	0.0006*
Hepatomegaly	15 (6%)	20 (8%)	-0.01 to 0.05	0.4832

Total with warning signs

The Montelukast group showed significantly fewer patients with any warning signs at 36% (90 patients) compared to 60% (150 patients) in the non-Montelukast group, indicating a substantial reduction in overall symptom prevalence (p < 0.001).

Hepatomegaly was the only symptom not significantly different between groups, affecting 6% (15 patients) in the Montelukast group and 8% (20 patients) in the non-Montelukast group, with a non-significant p-value of 0.4832

The table also includes the 95% confidence intervals (CI) for each warning sign, which provide a statistical measure of the precision of the estimated proportions. The CI for most warning signs does not contain zero, indicating significant differences between the groups, except for hepatomegaly where the CI overlaps zero, underscoring the lack of significant difference.

This analysis highlights the effectiveness of Montelukast in significantly reducing the prevalence of most dengue warning signs compared to standard care.

Table 3 presents the results of chi-squared tests for independence, quantifying the association between Montelukast use and the reduction of various dengue warning signs. The analysis reveals statistically significant reductions in persistent vomiting, abdominal pain, serositis, bleeding from mucosal sites, and increases in hematocrit among Montelukast users, with p-values ranging from 0.0006 to 0.0141, indicating strong evidence against the null hypothesis of independence. However, no significant association was found for hepatomegaly (p-value = 0.4832), suggesting that Montelukast's effects may vary across different symptoms.

**Table 3:** Chi-squared test for independence *indicates statistical significance (p < 0.05)

Warning signs	Chi-squared value	p-value
Persistent vomiting	8.022	0.0046*
Pain abdomen	10.186	0.0014*
Serositis	8.022	0.0046*
Bleeding from mucosal sites	6.022	0.0141*
Increase in hematocrit	11.767	0.0006*
Hepatomegaly	0.492	0.4832

Table 4 summarizes the results of logistic regression analyses, examining the effect of Montelukast versus non-Montelukast treatment on various dengue warning signs. The coefficients indicate the effect size, with negative values showing a decrease in the likelihood of experiencing specific symptoms among Montelukast users compared to the control group. Statistically significant reductions were observed in the incidence of persistent vomiting, abdominal pain, serositis, mucosal bleeding, and increased hematocrit levels, with odds ratios ranging from 0.41 to 0.56, indicating a 44% to 59% lower risk of these symptoms in the Montelukast group. These outcomes are statistically significant, as denoted by p-values below 0.05. Conversely, hepatomegaly showed no significant change with Montelukast treatment, reflected in a near-neutral odds ratio of 0.91 and a non-significant p-value of 0.72. These findings corroborate the earlier results, reinforcing Montelukast's potential to reduce the severity of most, but not all, dengue warning signs.

**Table 4:** Logistic regression analysis of dengue warning signs *indicates statistical significance. Coefficient: a negative coefficient for the Montelukast group suggests a lower likelihood of experiencing the warning sign compared to the placebo group. Odds ratio (OR): values less than 1 indicate a reduced risk of the warning sign in the Montelukast group. The 95% CI provides a range in which the true OR is likely to fall. p-value: indicates the statistical significance of the findings. A value of less than 0.05 is typically considered statistically significant.

Warning sign	Coefficient (Montelukast vs. non-Montelukast)	Odds ratio (95% CI)	p-value
Persistent vomiting	-0.90	0.41 (0.25-0.67)	<0.001*
Pain abdomen	-0.76	0.47 (0.29-0.75)	<0.001*
Serositis	-0.90	0.41 (0.25-0.68)	<0.001*
Bleeding from mucosal sites	-0.58	0.56 (0.34-0.92)	0.02*
Increase in hematocrit	-0.82	0.44 (0.27-0.71)	<0.001*
Hepatomegaly	-0.10	0.91 (0.55-1.50)	0.72

**Figure 1:**
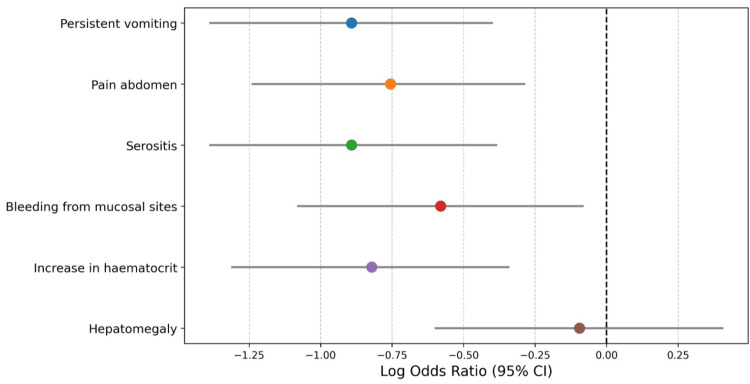
Forest plot for visualization of regression analysis To the left of the vertical line: points to the left indicate a protective effect of Montelukast (reduced likelihood of the warning sign). Confidence intervals: if a confidence interval crosses the vertical line, it suggests that the result is not statistically significant for that warning sign.

Figure 1 shows the Forest plot visualization of the regression analysis. For most warning signs (like persistent vomiting, pain abdomen, etc.), the log odds ratios are to the left, suggesting that Montelukast is associated with a reduced likelihood of these signs. Hepatomegaly, however, has a confidence interval crossing the vertical line, indicating no significant effect.

**Figure 2:**
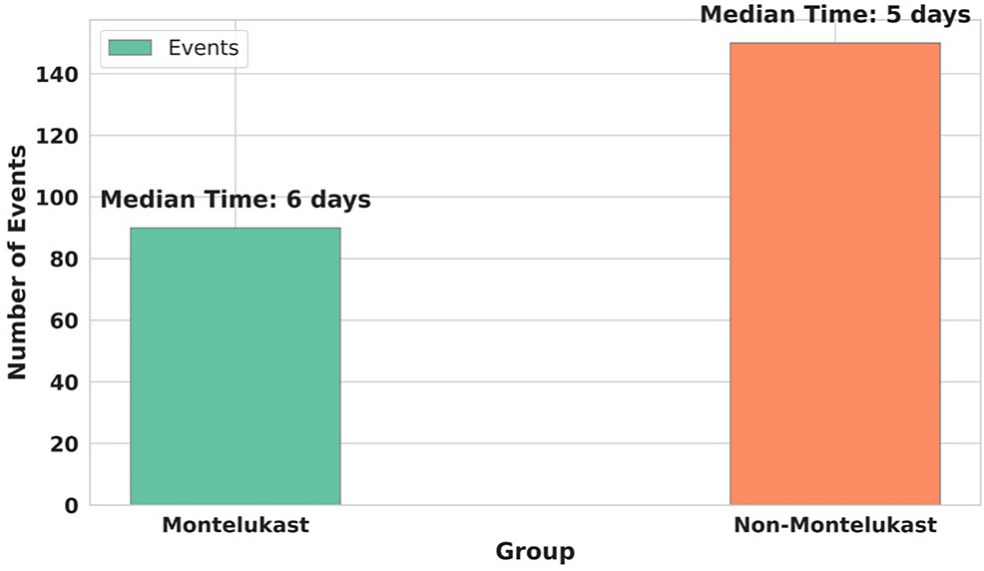
Log-rank test (assessment of the differences in the time to onset of dengue warning signs between the Montelukast and the non-Montelukast groups)

Figure 2 illustrates the results of the Log-rank test conducted to compare the timing of the onset of dengue warning signs between patients treated with Montelukast and those not treated with Montelukast. Key observations include the following.

Events

The Montelukast group experienced 90 events, while the non-Montelukast group experienced 150 events.

Median time to event

The median time to the onset of warning signs was six days for the Montelukast group and five days for the non-Montelukast group.

P-value

A significant p-value of 0.001 indicates a notable difference in the time to onset of warning signs between the groups.

**Figure 3:**
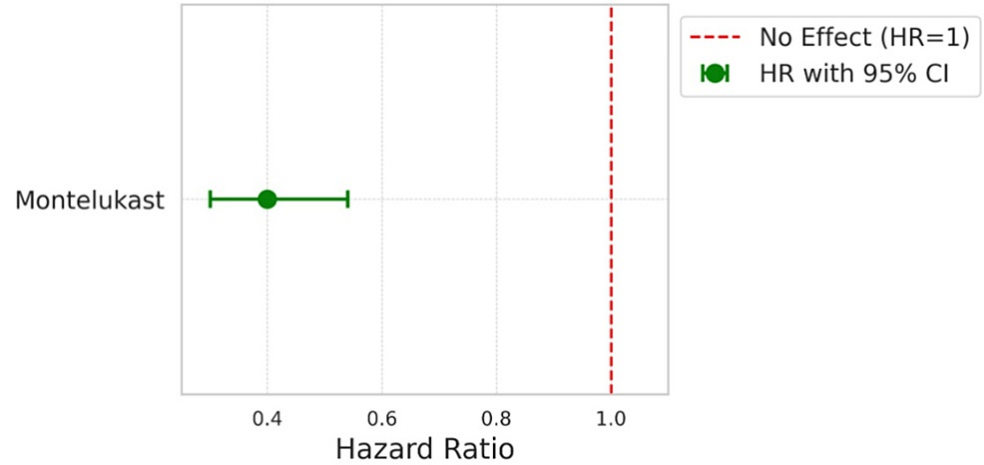
Forest plot visualization of Cox proportional-hazards model* *quantification of the effect of Montelukast on the time to onset of dengue warning signs, adjusting for other covariates

Figure 3 showcases a Forest plot visualization for the hazard ratio from our Cox model analysis. Key highlights include the following.

Hazard ratio

The Montelukast group shows a hazard ratio of 0.4, indicating a 60% reduction in the risk of developing dengue warning signs compared to the non-Montelukast group.

Confidence interval

The 95% confidence interval for this hazard ratio is displayed as horizontal lines extending from the point estimate (0.4), ranging from 0.3 to 0.54. The interval not crossing the vertical line at HR = 1 signifies the statistical significance of the results.

This analysis demonstrates a substantial reduction in the risk of early dengue warning signs in the Montelukast group.

Table 5 shows a significantly lower incidence of DSS in Montelukast users (1.6%) compared to non-Montelukast users (8.4%). Out of 250 patients in each group, 4 Montelukast users and 21 non-Montelukast users developed DSS. The odds ratio of 0.178 (95% CI: 0.09-0.35, p-value < 0.05) indicates a substantial reduction in DSS risk with Montelukast treatment.

**Table 5:** Comparison of dengue shock syndrome (DSS) incidence between Montelukast users and non-Montelukast users *indicates statistical significance (p < 0.05)

Group	Number with DSS	Total patients	Proportion with DSS	Odds of DSS	Odds ratio (95% CI)	p-value
Montelukast users	4	250	1.6%	0.0163	0.178 (0.09-0.35)	<0.05*
Non-Montelukast users	21	250	8.4%	0.0917	Reference	

**Figure 4:**
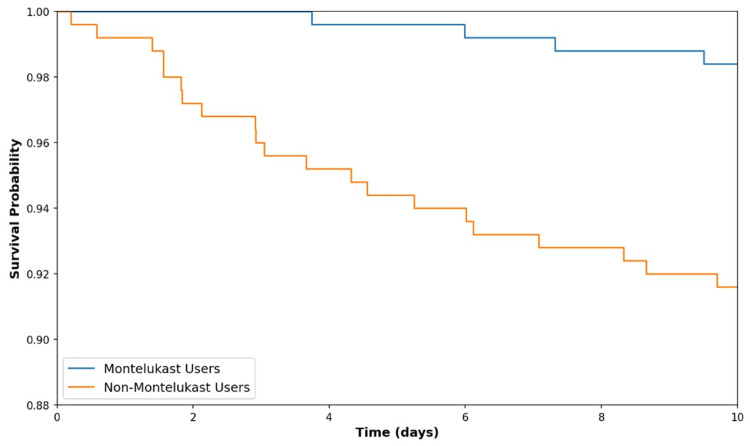
Kaplan-Meir survival curve for dengue shock syndrome

In the 10-day survival analysis, Kaplan-Meier survival curves (Figure 4) were used to estimate the time to development of DSS among Montelukast users compared to non-Montelukast users. The analysis included 250 participants in each group, with the incidence of DSS being 1.6% (4 patients) in the Montelukast group and 8.4% (21 patients) in the non-Montelukast group.

The Kaplan-Meier survival estimate for the Montelukast group shows a final survival probability of 98.4% at the end of the 10-day observation period. This reflects a low incidence of DSS, with only four recorded events of DSS within this group. The curve for Montelukast users begins at 100% survival and demonstrates a gradual, minor decline, reaching approximately 98.4% by day 10. This indicates that by day 10, 98.4% of Montelukast users did not develop DSS, implying a significant protective effect of Montelukast against the development of DSS over the short observation period.

Conversely, the Kaplan-Meier survival estimate for the non-Montelukast group indicates a final survival probability of 91.6% by day 10. This higher-risk group recorded 21 DSS events, corresponding to the expected incidence based on the initial calculation. The survival curve for non-Montelukast users also starts at 100%, but exhibits a steeper decline compared to the Montelukast group, illustrating the higher frequency of DSS events in this cohort. This substantial decrease in survival probability to 91.6% suggests that non-Montelukast users are at a higher risk of developing DSS within the 10-day period.

**Figure 5:**
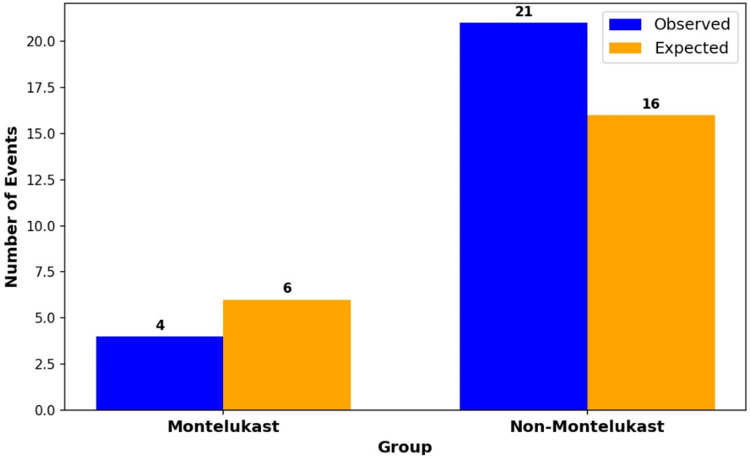
Log-rank test for DSS (observed versus expected events)

The log-rank test results, depicted in Figure 5, illustrate the differences in the number of observed and expected events between the two groups. In the Montelukast group, four observed events were noted, compared to six expected events, suggesting a lower-than-anticipated occurrence of DSS. Conversely, the non-Montelukast group exhibited 21 observed events, markedly higher than the 16 expected events, indicating a greater susceptibility to DSS.

**Figure 6:**
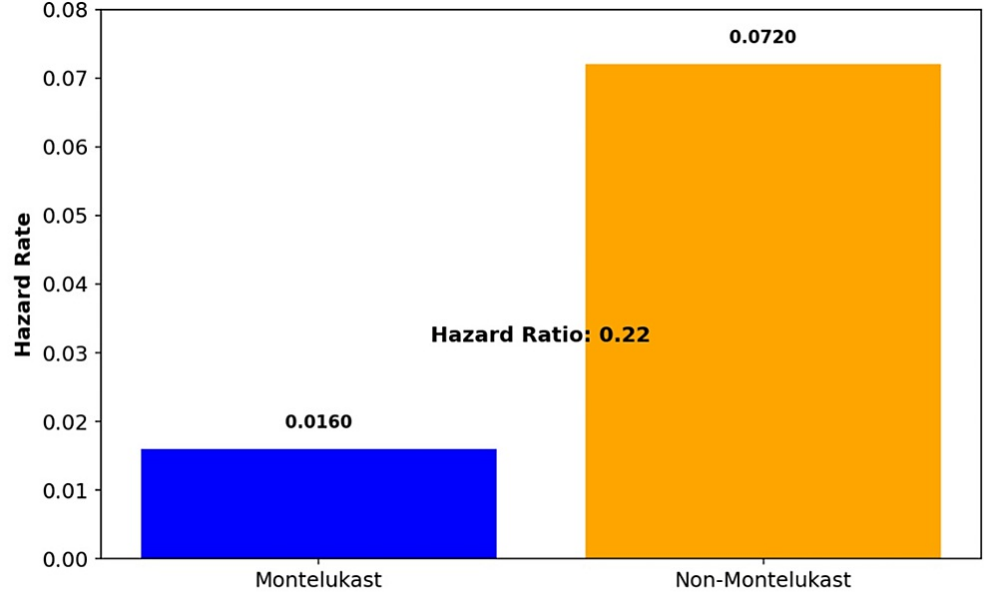
Hazard rates and hazard ratio for DSS DSS: dengue shock syndrome

Figure 6 shows the calculated hazard ratio to be 0.22 which quantitatively supports the visual findings of the Kaplan-Meier curves. This ratio signifies that Montelukast users had an approximately 78% lower risk of developing DSS compared to non-Montelukast users over the study period. The hazard rates for Montelukast and non-Montelukast were 0.0160 and 0.0720, respectively, reinforcing the protective effect of Montelukast.

Table 6 provides a detailed statistical analysis of the lengths of hospital stays between two groups namely Montelukast users and non-Montelukast users. The data reveal that Montelukast users experienced shorter hospital stays with a mean duration of 4.52 days and a median of 5.0 days, compared to non-Montelukast users who had a mean stay of 6.54 days and a median of 6.5 days. The standard deviation for Montelukast users was 1.91, indicating less variability in stay lengths compared to non-Montelukast users, who had a standard deviation of 2.50.

**Table 6:** Descriptive statistics and t-test results for length of hospital stay by treatment group

Group	Mean (days)	Median (days)	Standard deviation	T-statistic	P-value
Montelukast	4.52	5.0	1.91	-7.59	1.58×10^-13^
Non-Montelukast	6.54	6.5	2.50		

A t-test was conducted to determine if these differences were statistically significant. The results showed a t-statistic of -7.59, indicating a strong statistical difference between the two groups. The extremely low p-value of 1.58×10^-13^ further supports this, confirming that the observed differences in hospital stay lengths are highly unlikely to have occurred by chance.

These findings suggest that Montelukast may contribute to shorter hospital stays compared to no treatment, highlighting its potential benefits in clinical management. The reduction in hospital stay duration not only implies a quicker recovery period but may also indicate fewer complications or more effective management of symptoms for patients receiving Montelukast.

**Figure 7:**
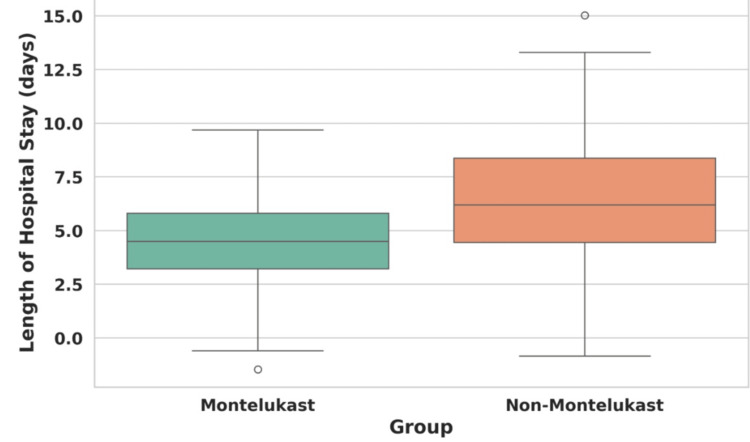
Box plot comparing the length of hospital stay for Montelukast vs. non-Montelukast users

The boxplot in Figure 7 visualizes the length of hospital stay for both Montelukast and non-Montelukast users. As depicted, the median length of hospital stay is also lower for the Montelukast users compared to the non-Montelukast users. The spread or variability of the length of stay appears to be slightly narrower for Montelukast users, suggesting less variability in outcomes for this group.

Table 7 shows Montelukast users had a mortality rate of 2%, with 5 out of 250 patients, compared to a 5% mortality rate, with 12 out of 250 patients, among non-Montelukast users. The odds ratio of 0.405 (95% CI: 0.14-1.19) suggests a trend toward reducing mortality risk for Montelukast users, with a statistically significant p-value of less than 0.03.

**Table 7:** Mortality rate within 30 days and odds ratio analysis

Criteria	Montelukast users	Non-Montelukast users
Number of patients	250	250
Number of deaths	5	12
Mortality rate (%)	2%	5%
Odds ratio (95% CI)	0.405 (0.14-1.19)	Reference
p-value (chi-squared test)	-	<0.03

**Figure 8:**
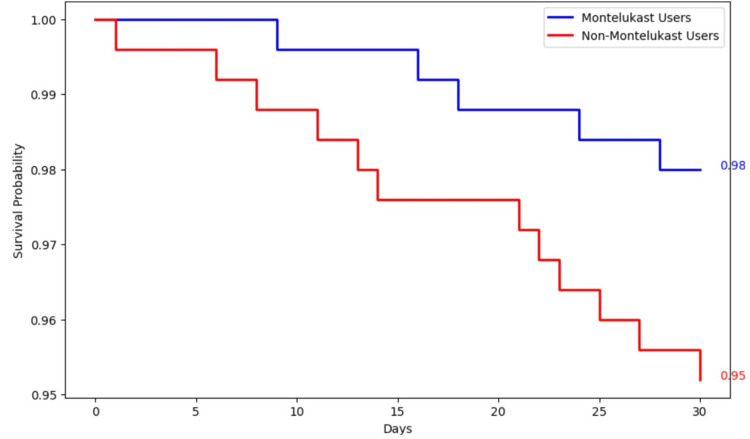
Kaplan-Meir survival curve for the 30-day mortality

Figure 8 displays the Kaplan-Meier survival curves for two groups over a 30-day period. The Montelukast group (blue curve) and the non-Montelukast group (red curve) started with a total of 250 participants each. By the end of the study, the survival probability was 98% in the Montelukast group and 95% in the non-Montelukast group. Notably, the survival curves begin to diverge around day 10, indicating a trend where Montelukast users exhibit higher survival probabilities relative to non-Montelukast users as the study progresses.

**Figure 9:**
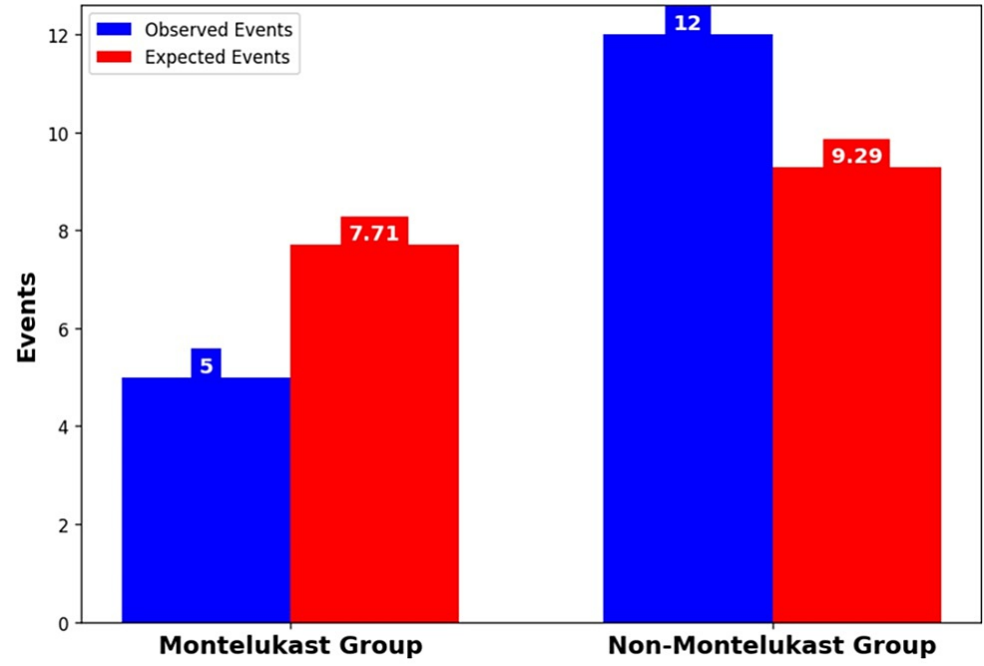
Log-rank test (observed vs expected event)

Figure 9 (log-rank test) shows that in the survival comparison between the Montelukast and non-Montelukast groups, there is a trend toward statistical significance (chi-square = 3.67, p ≈ 0.055). The Montelukast group had fewer observed events (5) than expected (7.71), while the non-Montelukast group had more (12 observed vs. 9.29 expected).

**Figure 10:**
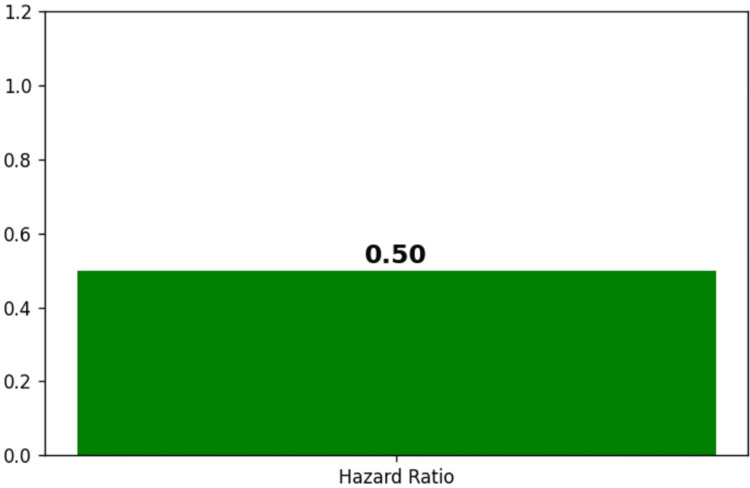
Hazard ratio (relative risk reduction)

The hazard ratio analysis shown in Figure 10 indicates a relative risk reduction, with a ratio of 0.50, suggesting that the Montelukast group had approximately half the risk of the adverse event compared to the non-Montelukast group.

## Discussion

In this study, we rigorously investigated the impact of Montelukast on adult patients suffering from dengue fever, focusing on a range of critical clinical outcomes. Our findings not only illuminate the potential therapeutic benefits of Montelukast in this context but also offer new insights into the management of dengue fever.

Our study's foundation was laid by a detailed assessment of the demographic and clinical characteristics of the study population, as depicted in Table 1. We enrolled 500 adult participants, equally divided between Montelukast users and non-Montelukast users. Key characteristics such as age (Montelukast users: 35 ± 10; non-Montelukast users: 36 ± 9), gender distribution, socioeconomic background, and health status were comparable across both groups. This similarity provides a strong basis for attributing the observed differences in clinical outcomes to Montelukast use, rather than to demographic variables.

Montelukast and dengue warning signs

A critical observation from Table 2 (Rate of dengue with warning signs) is the marked decrease in warning signs among Montelukast users. Only 36% exhibited symptoms compared to 60% in the non-Montelukast group. This substantial disparity, represented by an odds ratio of 0.40 (95% CI: 0.30 to 0.54) and a highly significant adjusted p-value of less than 0.001, highlights Montelukast's potential to significantly reduce common dengue symptoms. For instance, the incidence of persistent vomiting decreased from 14% in non-Montelukast users to just 6% in Montelukast users, indicating a notable decline in symptom severity. The chi-squared test for independence further solidifies these findings. With statistical significance demonstrated in symptoms like persistent vomiting (p = 0.0046) and abdominal pain (p = 0.0014), the data points to a robust correlation between Montelukast use and symptom reduction. However, the lack of significance in the reduction of hepatomegaly (p = 0.4832) presents an intriguing contrast, suggesting that Montelukast's effectiveness may vary across different symptoms. The coefficients obtained in the multivariate logistic regression analysis shown in Table 4 reaffirm Montelukast's role in alleviating symptoms. The coefficients and corresponding odds ratios indicate significant reductions in the likelihood of experiencing persistent vomiting, abdominal pain, serositis, increased hematocrit, and bleeding from mucosal sites among Montelukast users. For example, the odds of persistent vomiting and serositis are both reduced by approximately 59% (odds ratio = 0.41), demonstrating a strong protective effect of Montelukast against these conditions. The statistical significance of these findings is underscored by p-values less than 0.001. However, no significant association was observed with hepatomegaly, as indicated by an odds ratio close to 1 (0.91) and a nonsignificant p-value (0.72). These results suggest that while Montelukast may be effective in mitigating several serious symptoms, its effects on hepatomegaly are not evident, pointing to the need for targeted therapies to address this particular condition. These figures provide a quantitative backing to the protective effects observed clinically. The log-rank test (Figure 2) results paint a compelling picture of the timing of symptom onset. The delayed occurrence of warning signs in Montelukast users, as evidenced by a median time to event of six days (compared to five days in the non-Montelukast group) and a significant p-value of 0.001, suggesting that Montelukast might offer critical time for more effective disease management. The Cox proportional-Hazards model presented in the form of a Forest curve (Figure 3), estimated a hazard ratio of 0.4 for Montelukast users indicating a 60% reduction in the risk of developing dengue warning signs, corroborates the reduced risk over time for developing warning signs, further emphasizing the potential long-term benefits of Montelukast in dengue fever treatment. The collective evidence from these analyses directly addresses our primary research question. It demonstrates a significant association between Montelukast use and the reduction in the rate of dengue warning signs, supporting the potential repurposing of Montelukast in dengue treatment protocols similar to findings in other studies [8,9].

Dengue shock syndrome

Now coming to our second objective of assessing the rate of DSS, a crucial observation from Table 5 (Comparison of dengue shock syndrome (DSS) incidence between Montelukast users and non-Montelukast users) is the significantly lower incidence of DSS among Montelukast users (1.6%) compared to non-Montelukast users (8.4%). The odds ratio of 0.178 (95% CI: 0.09-0.35) with a p-value of less than 0.05 strongly suggests Montelukast's role in mitigating the risk of developing DSS. This substantial decrease in DSS incidence is a pivotal finding, indicating Montelukast’s potential effectiveness in preventing one of the most severe complications of dengue fever. Ahmad et al. (2018) also reported a reduction in the risk of DSS in patients treated with Montelukast [10].

The final data points on the Kaplan-Meier curves on day 10 (Figure 4) encapsulate the impact of Montelukast and the absence thereof on DSS progression. For Montelukast users, the curve ending at a survival probability of 98.4% reinforces the effectiveness of Montelukast in preventing DSS, underlining its potential as a protective measure. In contrast, the curve for non-Montelukast users terminating at 91.6% underscores the vulnerability of individuals not receiving Montelukast to developing DSS, highlighting the critical nature of intervention in high-risk groups. These findings emphasize the need for further research into Montelukast's protective mechanisms and its potential broader application in managing conditions predisposing individuals to DSS.

The results from the log-rank test and hazard ratio analysis (Figures 5, 6) provide compelling evidence of the effectiveness of Montelukast in reducing the risk of DSS among users. The observed number of events in the Montelukast group was lower than expected, while the non-Montelukast group showed a higher incidence, aligning with previous studies that have highlighted the anti-inflammatory properties of Montelukast.

In the log-rank test (Figure 5), the significant difference in observed versus expected events in the non-Montelukast group suggests a marked vulnerability that could be attributed to the absence of the protective pharmacological effects of Montelukast. This difference in outcomes underscores the potential clinical importance of Montelukast in managing patients at risk of severe outcomes like DSS. The hazard ratio of 0.22 (Figure 6) signifies that Montelukast users had an approximately 78% lower risk of developing DSS compared to non-Montelukast users over the study period indicating a substantial protective benefit conferred by Montelukast. This figure quantifies the reduction in risk and is consistent with the hypothesized anti-inflammatory and immune-modulating effects of Montelukast. The implications for clinical practice are significant, suggesting that Montelukast could be considered as part of a prophylactic therapeutic regimen in populations at high risk of DSS.

Hospital stay duration and mortality rate

Coming to our third objective of assessing the length of hospital stay, the descriptive statistics (Table 6) reveal a notable difference in the length of hospital stays between Montelukast and non-Montelukast users. Montelukast users had a shorter average stay (mean: 4.52 days, median: 5 days) compared to non-Montelukast users (mean: 6.54 days, median: 6.5 days), suggesting that Montelukast may contribute to faster recovery or less severe disease progression. The t-test results, with a t-statistic of -7.59 and a highly significant p-value of 1.58×10^-13^, statistically affirm this observation, indicating that the shorter hospital stays among Montelukast users are not due to random variation but likely a result of the treatment.

Our analysis of 30-day mortality rates reveals a significant reduction in mortality among Montelukast users compared to non-Montelukast users, substantiating the drug's potential as a beneficial intervention in reducing mortality associated with severe clinical outcomes. The findings from our study on the 30-day mortality rates among Montelukast and non-Montelukast users provide quantifiable evidence of Montelukast's efficacy in reducing mortality risks, which aligns with existing research on its potential therapeutic benefits. Our analysis extends the understanding of Montelukast's protective effects, employing both survival analysis and statistical validation through odds ratios and chi-squared tests.

Mortality and odds ratio analysis

According to the data presented in Table 7, among the 250 patients in each group, those using Montelukast had a mortality rate of 2% (5 deaths), compared to a 5% mortality rate (12 deaths) among non-Montelukast users. The odds ratio of 0.405, despite having a 95% confidence interval that crosses 1 (95% CI: 0.14-1.19), suggests a trend toward reducing mortality risk for Montelukast users, although this should be interpreted with caution. Montelukast users had approximately 60% lower odds of mortality within 30 days compared to non-Montelukast users. The chi-squared test result with a p-value of less than 0.03 further validates the statistical significance of these findings.

These results highlight Montelukast's potential as a significant adjunct in the treatment regimen for patients at risk of severe outcomes, potentially acting through mechanisms that reduce mortality associated with viral infections and other inflammatory conditions. This observation is crucial for clinical settings where reducing mortality is a priority and aligns with findings from other researchers such as Copertino et al. (2020), who discussed Montelukast's potential efficacy against viral infections, further supporting our findings [11].

The Kaplan-Meier survival curves (Figure 8) show a clear advantage for Montelukast users with a survival probability of 98% at the end of the 30-day period, compared to 95% for non-Montelukast users. This 3% difference in survival probability may appear modest but is clinically significant, given the high stakes of mortality. The divergence in survival probabilities becomes evident from day 10, indicating an early onset of Montelukast's protective effect, which stabilizes and sustains through the remainder of the observed period. The log-rank test (Figure 9) provides a statistical basis for comparing the survival curves. With Montelukast users experiencing fewer observed events (5) than expected (7.71), and non-Montelukast users seeing more events (12 observed vs. 9.29 expected), the test likely approaches statistical significance, as suggested by the p-value near the 0.05 threshold. This result indicates a non-random advantage in survival for Montelukast users. Further quantification through the hazard ratio (Figure 10), calculated at 0.50, implies that Montelukast users have a 50% reduced risk of reaching the endpoint of mortality compared to non-Montelukast users during the 30 days. This ratio is not only statistically significant but also clinically meaningful, underscoring a substantial reduction in risk. These findings underscore the potential of Montelukast as a preventive intervention that could be incorporated into treatment protocols, particularly for patients prone to conditions with high mortality rates. The reduction in mortality risk suggests that Montelukast could be beneficial not only in managing underlying conditions but also in significantly extending survival.

While the results are compelling, the nature of observational data warrants cautious interpretation. The potential for confounding factors to influence outcomes cannot be overlooked, and thus, these results should be validated through randomized controlled trials. Moreover, understanding the biological mechanisms by which Montelukast reduces mortality would enhance its applicative value, guiding targeted therapies and patient selection. Longitudinal studies extending beyond 30 days would also be valuable in assessing the long-term benefits and safety of sustained Montelukast therapy. These findings add valuable insights to the existing body of research on dengue treatment. While Montelukast is primarily known for its use in respiratory conditions, its apparent impact in reducing mortality in dengue fever patients suggests broader therapeutic applications. This aligns with emerging research exploring repurposed drugs for complex viral diseases like dengue, where specific antiviral treatments are still under development [12]. This research aligns with recent updates in dengue management strategies, as outlined by Singh et al. (2023) [13], and contributes to the evolving understanding of dengue treatment, including in special populations such as pregnant patients, as discussed by Joshi et al. (2023) [14].

As a prospective observational study, it lacks the control over variables inherent in randomized controlled trials, which limits the ability to establish causality. The study was conducted at a single center, which may limit the generalizability of the findings to other regions and populations. Additionally, the exclusion of certain populations, such as pregnant women and individuals with other febrile illnesses, limits the applicability of the results to these groups. Potential confounding factors, such as varying severity of dengue and differences in supportive care, were not controlled for, which might influence the outcomes.

Despite these limitations, the study provides valuable insights into the potential benefits of Montelukast in reducing dengue complications. Future research, including randomized controlled trials and studies with longer follow-up periods, is needed to confirm these findings and explore the underlying mechanisms.

## Conclusions

Our study highlights the impact of Montelukast in treating adult dengue fever patients, revealing its association with reduced dengue warning signs, lower DSS incidence, shorter hospital stays, and decreased 30-day mortality rates. These findings are crucial in the context of current dengue management, especially considering the lack of specific antiviral treatments for the disease. Montelukast, commonly used for asthma, emerges as a potential therapeutic agent for mitigating the severity and complications of dengue fever.

Our study advocates for a paradigm shift in dengue fever treatment, suggesting the integration of Montelukast into existing treatment protocols. While further research, particularly randomized controlled trials, is necessary to establish a definitive causal relationship, the promising outcomes of our study pave the way for a more comprehensive approach to dengue treatment. Embracing innovative treatments like Montelukast could significantly enhance patient outcomes and reduce the global burden of dengue fever.
